# Investigation of Contrast-Induced Neurotoxicity and the Effects of Sildenafil and *N*-Acetylcysteine on HIF-1α Levels in Wistar Rats

**DOI:** 10.3390/brainsci16040362

**Published:** 2026-03-27

**Authors:** Ismail Altintop, Mehmet Tatli, Zeynep Soyer Sarica, Arzu Hanım Yay, Çiğdem Karakukcu

**Affiliations:** 1 Department of Emergency Medicine, Kayseri City Medical Faculty, Health Science University, Kayseri 38090, Türkiye; 2 Department of Emergency Medicine, Van Training and Research Hospital, Health Science University, Van 65100, Türkiye; 3 Central Research Laboratory Application and Research Center, Gebze Technical University, Kocaeli 41400, Türkiye; 4 Department of Histology and Embryology, School of Medicine, Erciyes University, Kayseri 38280, Türkiye; 5 Department of Biochemistry, School of Medicine, Erciyes University, Kayseri 38280, Türkiye

**Keywords:** phosphodiesterase-5 inhibitors, oxidative stress, neuroprotection, contrast media-induced encephalopathy

## Abstract

**Highlights:**

**What are the main findings?**

- Contrast media administration induced a significant increase in HIF-1α immunoreactivity in a rat model of contrast-induced neurotoxicity.
- The primary biochemical endpoint (tissue HIF-1α by ELISA) did not reach statistical significance across groups.
- Secondary IHC analysis suggests a potential attenuation of HIF-1α expression in the sildenafil-treated group, warranting confirmation in adequately powered studies.

**What are the implications of the main findings?**

- HIF-1α immunoreactivity may serve as a potential biomarker for identifying contrast-induced neural injury, pending further validation.
- These preliminary findings support further investigation of PDE5 inhibitors as candidate neuroprotective agents in contrast-induced encephalopathy.

**Abstract:**

Background: Contrast-induced encephalopathy (CIE) is an uncommon yet clinically significant complication associated with iodinated contrast media, with its mechanisms remaining unclear. Objective: The aims of this study are to examine the neurotoxic effects of contrast media and assess the neuroprotective roles of *N*-Acetylcysteine (NAC) and sildenafil with regard to HIF-1α expression. Methods: Thirty-six female Wistar albino rats (*n* = 36) were allocated into four experimental groups (*n* = 9 each): control, contrast media + saline (CMA + Saline), contrast media + NAC (CMA + NAC), and contrast media + sildenafil (CMA + Sildenafil). NAC (150 mg/kg) and sildenafil (50 mg/kg/day) were administered intragastrically for 48 h before exposure to contrast media. Biochemical, histopathological, and immunohistochemical evaluations were conducted 48 h post-contrast administration. Results: Exposure to contrast media resulted in neuronal death, vascular obstruction, and increased hypoxia-inducible factor-1 alpha (HIF-1α) immunoreactivity. The primary outcome measure, tissue HIF-1α concentration by ELISA, did not differ significantly among groups (*p* = 0.119). Semi-quantitative immunohistochemical analysis revealed significant group differences in HIF-1α immunoreactivity (*p* < 0.001), with all injury/treatment groups differing significantly from control. The difference between the contrast media group and the sildenafil-treated group approached but did not reach statistical significance after correction for multiple comparisons (Dunn’s test, *p* = 0.050). Conclusions: The primary biochemical endpoint did not demonstrate significant group differences. Secondary IHC analysis suggests a potential attenuation of HIF-1α immunoreactivity by sildenafil, though this did not reach statistical significance and requires confirmation in adequately powered studies. HIF-1α immunoreactivity warrants further investigation as a potential biomarker for contrast-induced neural injury.

## 1. Introduction

Contrast media are extensively utilized in diagnostic and therapeutic endovascular procedures but may induce contrast-induced encephalopathy (CIE), an acute, reversible neurological disorder [1,2,3]. CIE is infrequently diagnosed due to its intricate pathophysiology and nonspecific manifestations, which may include motor or sensory disturbances, vision impairment, confusion, and seizures, generally subsiding within 24–48 h [3,4,5,6].

There are many ways that CIE can start. A crucial step involves temporary disruption of the blood–brain barrier (BBB), facilitating the entry of neurotoxic contrast material into the brain parenchyma and resulting in vasogenic edema [1,2,4,7]. Contrast agents with elevated osmolarity can directly harm endothelial cells, and contrast-induced vasoconstriction may lead to localized ischemia and hypoxia [4,7,8].

Contrast agents produce reactive oxygen species (ROS) via various mechanisms, resulting in significant oxidative damage to cellular macromolecules and rendering neurons susceptible to injury and apoptosis [1,3,4,9,10]. Cellular hypoxia and oxidative stress induce the activation of hypoxia-inducible factor-1 (HIF-1), which serves as a principal regulator of the cellular response to hypoxia [11,12]. Furthermore, hypoxia-inducible factor 1-alpha (HIF-1α) is a key transcription factor that acts as a master regulator of cellular responses to hypoxic conditions [10]. Acute activation of HIF-1α enhances neuronal survival during cerebral ischemia and stroke; however, prolonged excessive expression may lead to neuroinflammation [11,12,13].

*N*-Acetylcysteine (NAC), a strong antioxidant and precursor to glutathione, has been shown to protect the brain from damage by lowering oxidative stress and inflammation in different models of brain injury [14,15,16,17]. Sildenafil, a selective phosphodiesterase-5 (PDE-5) inhibitor, enhances cerebral blood flow and stimulates various pro-survival signaling pathways, potentially influencing HIF-1α signaling via cGMP-dependent mechanisms [18,19,20].

Although contrast-induced encephalopathy (CIE) holds clinical importance, direct experimental evidence regarding the effects of *N*-Acetylcysteine (NAC) and sildenafil on contrast-induced brain injury and HIF-1α signaling remains scarce. Therefore, this study sought to clarify the mechanisms responsible for contrast-induced neurotoxicity and assess the neuroprotective efficacy of NAC and sildenafil.

## 2. Materials and Methods

### 2.1. Animals and Ethical Approval

The Erciyes University Experimental Research and Application Center (14 June 2017; 17/063) approved all animal procedures. Thirty-six 16-week-old female Wistar rats (200–250 g) were obtained from the Erciyes University Experimental Research Center. The rats were maintained under controlled conditions (23 ± 2 °C, 50 ± 5% humidity, 12 h light/dark cycle) with commercial feed (Purina, Düzce, Türkiye) and water provided ad libitum. Animals were acclimatized to these conditions for seven days prior to experimental procedures. Inclusion and Exclusion Criteria: The following inclusion criteria were established a priori: (1) healthy female Wistar rats aged 16 weeks; (2) body weight within a 200–250 g range; and (3) normal baseline health status confirmed during the acclimatization period.

Exclusion criteria included: (1) animals showing signs of illness, distress, or abnormal behavior prior to or during the experiment; (2) body weight outside the 200–250 g range; (3) death during experimental procedures; and (4) technical failures during sample collection or laboratory analysis (e.g., hemolysis, inadequate tissue preservation).

No animals met the exclusion criteria during this study. All 36 rats completed the experimental protocol, and no data points were excluded from the statistical analyses. The rats were housed under controlled conditions (23 ± 2 °C, 50 ± 5% humidity, 12 h light/dark cycle) with a sufficient amount of commercial feed produced by Purina (Düzce, Türkiye) and water provided ad libitum.

### 2.2. Experimental Design

Randomisation was performed using a computer-generated random number sequence (SPSS 22.0, SPSS Inc., Chicago, IL, USA). Each rat was assigned a unique identification number, and these numbers were randomly allocated to one of four experimental groups. To minimize potential confounding factors, all experimental procedures were conducted at consistent times of day (between 09:00 and 12:00), cage positions were rotated weekly within the animal room, and all animals were handled by the same trained personnel throughout the study. Nine rats were randomly allocated into one of four groups (*n* = 9 each): Group A (Control, saline only), Group B (CMA + Saline, contrast media administration followed by saline), Group C (CMA + NAC, contrast media administration followed by *N*-Acetylcysteine), and Group D (CMA + SIL, contrast media administration followed by sildenafil). Abbreviations used hereafter: CMA, contrast media administration; NAC, *N*-Acetylcysteine; SIL, sildenafil. NAC was sourced from Basel Pharmaceutical Co., Ltd. (Istanbul, Türkiye), sildenafil was sourced from Actavis Pharmaceutical Co., Ltd. (Istanbul, Türkiye), and the low-osmolar, nonionic contrast media agent (iopromide) was sourced from Opakim Pharmaceutical Co., Ltd. (Istanbul, Türkiye). NAC (150 mg/kg) and sildenafil (50 mg/kg/day) were administered intragastrically for 48 h prior to contrast exposure. Group A received only isotonic serum throughout the experiment, and Group B received isotonic serum for 48 h before contrast administration. All groups received an anesthetic dosage of 60 mg/kg of pentobarbital prior to the induction of CMA, which was achieved via drug injection into a tail vein. Pentobarbital was selected for the contrast media administration phase to provide hemodynamic stability during intravenous injection, while ketamine/xylazine was used for terminal tissue collection to provide surgical-plane anesthesia. The potential confounding effects of different anesthetic agents on HIF-1α expression and cerebrovascular physiology are acknowledged as a limitation (see Section 4.4). Groups B, C, and D received iopromide (1600 mg iodine/kg) at time zero [21]. The intravenous dose of iopromide (1600 mg iodine/kg) was selected based on previously published experimental models of contrast-induced neurotoxicity in rodents. Wible et al. reported an intracisternal LD50 of 122 mg iodine/kg for iopromide in rats, highlighting its considerable neurotoxic potential [21]. As intravenous administration requires substantially higher doses to overcome the blood–brain barrier and produce consistent neurotoxic effects, the dose used in the present study was calibrated accordingly, in line with the range employed by Doerfler et al., who administered intravenous iopromide at doses up to 1036 mg iodine/kg in a rat model [22].

Animal Welfare Monitoring: To minimize pain and distress, all invasive procedures were performed under deep anesthesia using ketamine (90 mg/kg) and xylazine (10 mg/kg) administered intraperitoneally. Adequate depth of anesthesia was confirmed by the absence of pedal withdrawal reflex before any procedures.

Humane Endpoints: The following humane endpoints were established prospectively: (1) weight loss exceeding 20% of baseline body weight; (2) inability to reach food or water; (3) severe respiratory distress; (4) complete immobility or inability to right when placed on side; (5) seizures or severe neurological signs; (6) signs of severe unrelieved pain or distress. Animals meeting any of these criteria would be immediately euthanized.

Monitoring: Animals were monitored twice daily (morning and evening) for clinical signs including body weight changes, food and water intake, posture, mobility, respiratory pattern, and behavioral abnormalities. Additional monitoring was performed immediately following contrast agent administration.

Adverse Events: No animals reached humane endpoint criteria during this study. No mortality, abnormal behavior, or unexpected adverse events occurred throughout the experimental period. All 36 rats completed the study protocol without complications. All rat groups were maintained under standard nutritional conditions following the procedure. After a 48 h period, tissue samples were collected from rats under ketamine/xylazine anesthesia, in accordance with the CMA administration time. All groups were sacrificed simultaneously to ensure consistency.

**Figure 1:**
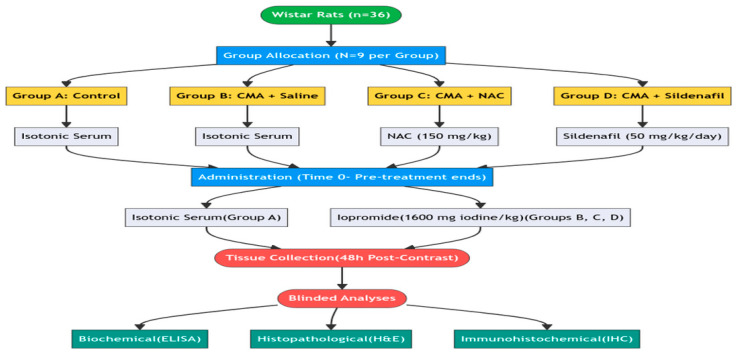
Flowchart of the study design.

Blinding procedures: (1) Allocation: Group allocation was performed by a researcher not involved in outcome assessment. (2) Conduct of experiment: The investigator administering treatments was not blinded due to the distinct nature of the interventions. (3) Outcome assessment: Tissue and serum samples were coded with numerical identifiers by an independent technician before analysis. All ELISA measurements and immunohistochemical evaluations were performed by investigators blinded to group allocation. (4) Data analysis: Statistical analysis was conducted on coded datasets, and unblinding occurred only after all analyses were completed. The experimental design and chronological sequence of the study are illustrated in the flow chart provided in Figure 1.

### 2.3. Biochemical Analysis of Tissue

Cerebral tissue samples from rats were excised with weights standardized to 0.25 g. Subsequently, frozen tissues and 1 mL of phosphate-buffered saline (pH 7.4) were introduced into screw-cap 2.0 mL tubes containing 0.4 g of sterile zirconium beads (comprising 0.3 g of 0.1 mm beads and 0.1 g of 0.5 mm beads). The tubes were inserted into a 24 microtube homogenizer (BeadBug™ D2400 BeadBlaster, Sayreville, NJ, USA) and subjected to processing for 1 min, comprising 6 cycles with 30 s intervals, at a speed of 6.5 m/s. Next, the tubes were incubated in a cold nitrogen tank for 3 min, followed by a repetition of the same process using the homogenizer, and then underwent centrifugation at 4 °C and 16,000× *g* for 10 min. Supernatants were then aliquoted into a new 2.0 mL tube for subsequent analysis. The primary outcome measure of this study was tissue HIF-1α concentration quantified by enzyme-linked immunosorbent assay (ELISA). This biomarker was selected as the primary endpoint because it directly reflects the hypoxic cellular response central to our hypothesis regarding contrast-induced neurotoxicity, and it was used to inform the sample size calculation.

Secondary outcome measures included: (1) serum HIF-1α concentration measured by ELISA, and (2) semi-quantitative assessment of HIF-1α immunoreactivity intensity in brain tissue sections by immunohistochemistry.

Tissue HIF-1α levels were quantified using a commercially available kit employing the quantitative sandwich enzyme immunoassay technique (Rat [HIF-1α] ELISA kit; SunRed Biotechnology Company, Shanghai, China).

### 2.4. Histopathological Assessment

Brain tissue was extracted for biochemical and histopathological analyses. Tissues from all groups were fixed in a 10% formaldehyde solution for 48 h, dehydrated using a graded alcohol series, and cleared with xylene for examination via light microscopy. Following dehydration, the brain tissues were embedded in paraffin. Then, 5 μm sections were obtained from the paraffin blocks using a microtome and subsequently placed on poly-lysine slides. All groups underwent hematoxylin–eosin (H&E) staining to provide a morphological overview of the tissue and its structure. Following staining, the sections were examined using an Olympus BX-51 light microscope (Olympus BX-51, Tokyo, Japan) and images were captured.

### 2.5. Immunohistochemistry

Immunohistochemistry: Immunohistochemical staining utilizing the avidin–biotin method was conducted to identify immunopositive cells expressing HIF-1α. Sections derived from paraffin blocks were incubated for two hours at 60 °C. Subsequently, the slides were placed in xylene for deparaffinization (2 changes, 5 min each), followed by rehydration through descending grades of ethanol (100%, 95%, 70%) to distilled water. Following rinsing in distilled water, a 3% hydrogen peroxide (H_2_O_2_) solution was applied for 12 min to inhibit endogenous peroxidase activity. The slides then underwent two washes in phosphate-buffered saline (PBS) at pH 7.4, each lasting 5 min.

Antigen Retrieval: For heat-induced epitope retrieval, sections were immersed in citrate buffer (pH 6.0) and heated in a microwave oven at 95–98 °C. This process reverses formaldehyde-induced protein cross-linking, thereby exposing antigenic epitopes that were masked during fixation. Slides were then cooled to room temperature in the citrate buffer.

Immunostaining: The procedure was executed sequentially using the Anti-Polyvalent HRP kit (Thermo Scientific, Waltham, MA, USA). The slides underwent two washes with PBS, each lasting 5 min, followed by treatment with Ultra V Block for 10 min to inhibit nonspecific binding. Following the blocking step, sections were incubated with rabbit anti-HIF-1α primary antibody (1:100, sc-53546, Santa Cruz Biotechnology, Dallas, TX, USA) overnight at 4 °C without prior washing. For negative controls, phosphate-buffered saline (PBS) was utilized in place of the primary antibody.

The slides underwent incubation for 1 h at room temperature, followed by two washes with PBS (5 min each). Subsequently, a biotinylated secondary antibody was applied for 10 min. Following a subsequent wash with PBS, the tissues were incubated in the streptavidin–peroxidase enzyme complex for 10 min. The slides were then washed with PBS and treated with diaminobenzidine (DAB) chromogen (DAB Plus substrate system) for 1–10 min to visualize immunoreactivity. Sections were rinsed with distilled water, counterstained with Gill’s hematoxylin, and mounted with a clear mounting medium.

Quantification: Sections were analyzed using a light microscope (Olympus BX51, Tokyo, Japan). Ten random visual fields were captured from each section at ×400 magnification. Immunopositive cells were quantified using ImageJ Software v1.53h version (National Institutes of Health, Bethesda, MD, USA) by a blinded investigator. Cytoplasmic immunoreactivity for HIF-1α was evaluated in the frontoparietal cortex (layers II–VI) of all experimental animals at standardized anteroposterior levels (bregma −1.0 to −3.0 mm).

### 2.6. Statistical Analysis

The Shapiro–Wilk normality test and Q–Q graphs were utilized to assess the normality of the data. Data are presented as counts for categorical variables, and as mean ± SD or median (25–75th percentile) for continuous variables. Group comparisons were conducted using the Kruskal–Wallis test for non-normally distributed data, followed by Dunn’s test with Bonferroni correction for pairwise comparisons where the omnibus test was significant. One-way analysis of variance was used for normally distributed data. Calculations and power analysis were carried out using specialized statistical software, including SPSS 22.0 (SPSS Inc., Chicago, IL, USA) and MINITAB 17 (Minitab Inc., State College, PA, USA). A *p*-value below 0.05 was considered statistically significant. Sample size was calculated based on preliminary data from our laboratory using G*Power 3.1 (α = 0.05, power = 0.80, f = 0.585, k = 4), yielding *n* = 9 per group. Effect sizes were calculated as epsilon-squared (ε^2^) for the Kruskal–Wallis omnibus test and Cohen’s d and rank-biserial correlation (r) for pairwise comparisons, providing measures of the practical significance of the observed differences. The power analysis indicated that a minimum of 9 animals per group would be required to detect a clinically meaningful difference with adequate statistical power. No additional animals beyond this minimum were used to adhere to the 3Rs principle (Replacement, Reduction, Refinement) of ethical animal research.

## 3. Results

No fatalities were observed among the rats involved in this study, and no significant anomalies were observed in the nutrition or activity of the rats across the groups. HIF-1α concentrations and immunoreactivity parameters were measured for all groups and compared among Group A, Group B, Group C, and Group D, as presented in Table 1.

### 3.1. Comparison of HIF-1α Levels and Immunohistochemical Findings

In Group A, HIF-1α and related parameters were evaluated. The effects of NAC and SIL treatments on rats are summarized in Table 1, as well as the HIF-1α levels in brain tissues.

The comparison of brain tissue HIF-1α concentrations (ng/mL) did not differ significantly among groups (*p* = 0.121). The Kruskal–Wallis test revealed a statistically significant difference in IHC-IPC among groups (H(3) = 102.22, *p* < 0.001, ε^2^ = 0.279, large effect). Post hoc pairwise comparisons using Dunn’s test with Bonferroni correction demonstrated that all three injury/treatment groups differed significantly from control (all *p* < 0.001). However, comparisons among treatment groups did not reach statistical significance: Group B vs. Group D (*p* = 0.050), Group B vs. Group C (*p* = 1.000), and Group D vs. Group C (*p* = 0.195). The comparison between Group B (CMA) and Group D (CMA + SIL) yielded a borderline *p*-value with a medium effect size (Cohen’s d = 0.71), suggesting a trend that may reach significance with greater statistical power (Table 1).

### 3.2. Histological Findings

**Figure 2:**
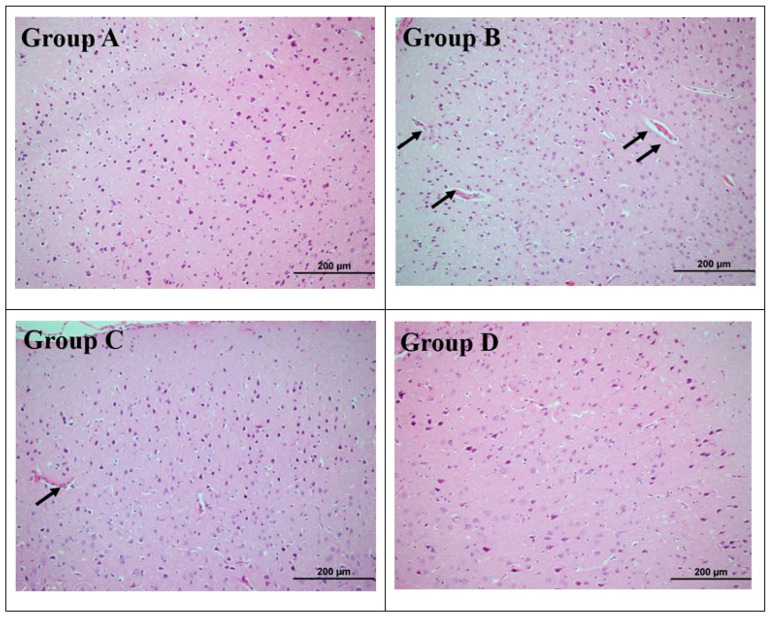
Histopathological findings (H&E staining, magnification: 100 µm). The nucleus and cytoplasm appear blue and pink, respectively. Arrows indicate vascular congestion. Group A (Control) shows normal histological architecture with well-preserved neuronal cell bodies. Group B (CMA + Saline) exhibits vascular congestion in both gray and white matter. Group C (CMA + NAC) demonstrates mild pathological alterations, primarily vascular congestion, indicating partial tissue protection. Group D (CMA + SIL) displays largely preserved and normal histoarchitectural features, closely resembling the control group.

The hematoxylin–eosin (H&E) staining technique was used to visualize the nuclei and cytoplasm of cells for histological evaluation. Under H&E staining, nuclei appear blue while the cytoplasm is stained pink, as shown in Figure 2. Analysis of brain sections demonstrated normal histological architecture with well-preserved neuronal cell bodies in the control group. Microscopic examination of Group B brain tissue revealed vascular congestion in both gray and white matter. In the NAC-treated group (Group C), only mild pathological alterations—primarily vascular congestion—were observed in comparison to Group A. Conversely, brain tissues from Group D (CMA + SIL) showed largely preserved and normal histoarchitectural features.

### 3.3. Immunohistochemical Observations

**Figure 3:**
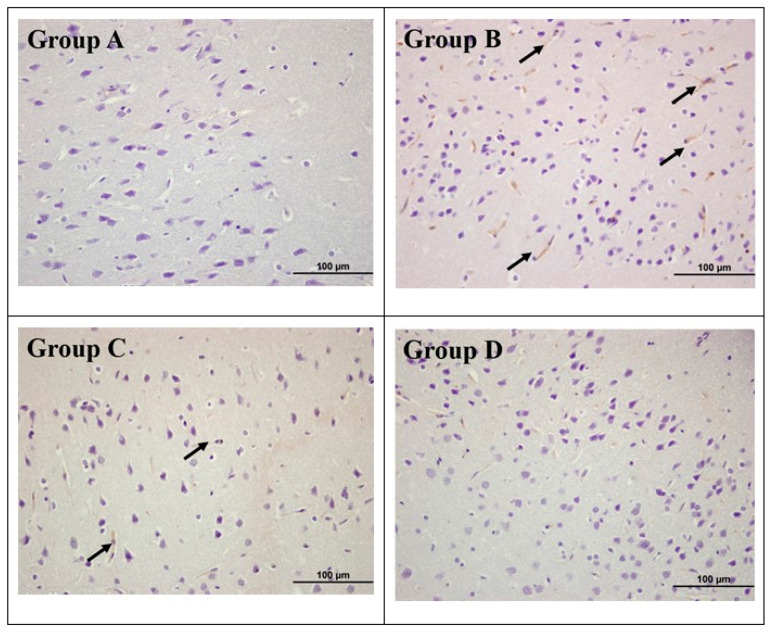
Immunohistochemical evaluation of brain tissues across experimental groups (magnification: 100 µm). Sections were stained with HIF-1α primary antibody (arrow; HIF-1α + cells), and HIF1α levels were compared statistically between groups with the number of positive cells. Group A (Control) shows normal neuronal morphology with no significant immunoreactivity. Group B (CMA + Saline) exhibits intense positive staining and significant neuronal damage (indicated by arrows), representing the neurotoxic effect of the contrast media. Group C (CMA + NAC) shows a moderate reduction in positive staining and cellular injury compared to Group B, indicating partial protection by *N*-Acetylcysteine. Group D (CMA + SIL) demonstrates the most significant reduction in immunoreactivity and a marked preservation of neuronal structure, closely resembling the control group and supporting the superior protective effect of sildenafil.

Figure 3 shows brain sections stained with the HIF-1α primary antibody. Cytoplasmic immunoreactivity was evaluated in the cortical regions of all experimental animals.

No mortality or abnormal behavior was observed. Although ELISA analysis indicated no significant differences in HIF-1α levels among groups (*p* = 0.119), immunohistochemical analysis revealed highly significant differences in HIF-1α immunoreactivity (*p* < 0.001), with highest expression in Group B (CMA + Saline) and lowest in Group D (CMA + SIL), while Group C (CMA + NAC) showed intermediate levels.

Histopathological examination revealed normal architecture in control tissues, while Group B showed significant vascular congestion and neuronal degeneration, Group C showed mild pathological alterations with reduced neuronal injury, and Group D showed preserved cortical architecture, which was nearly indistinguishable from that of the control, with minimal neuronal degeneration and vascular congestion.

#### HIF-1α Immunoreactivity

**Figure 4:**
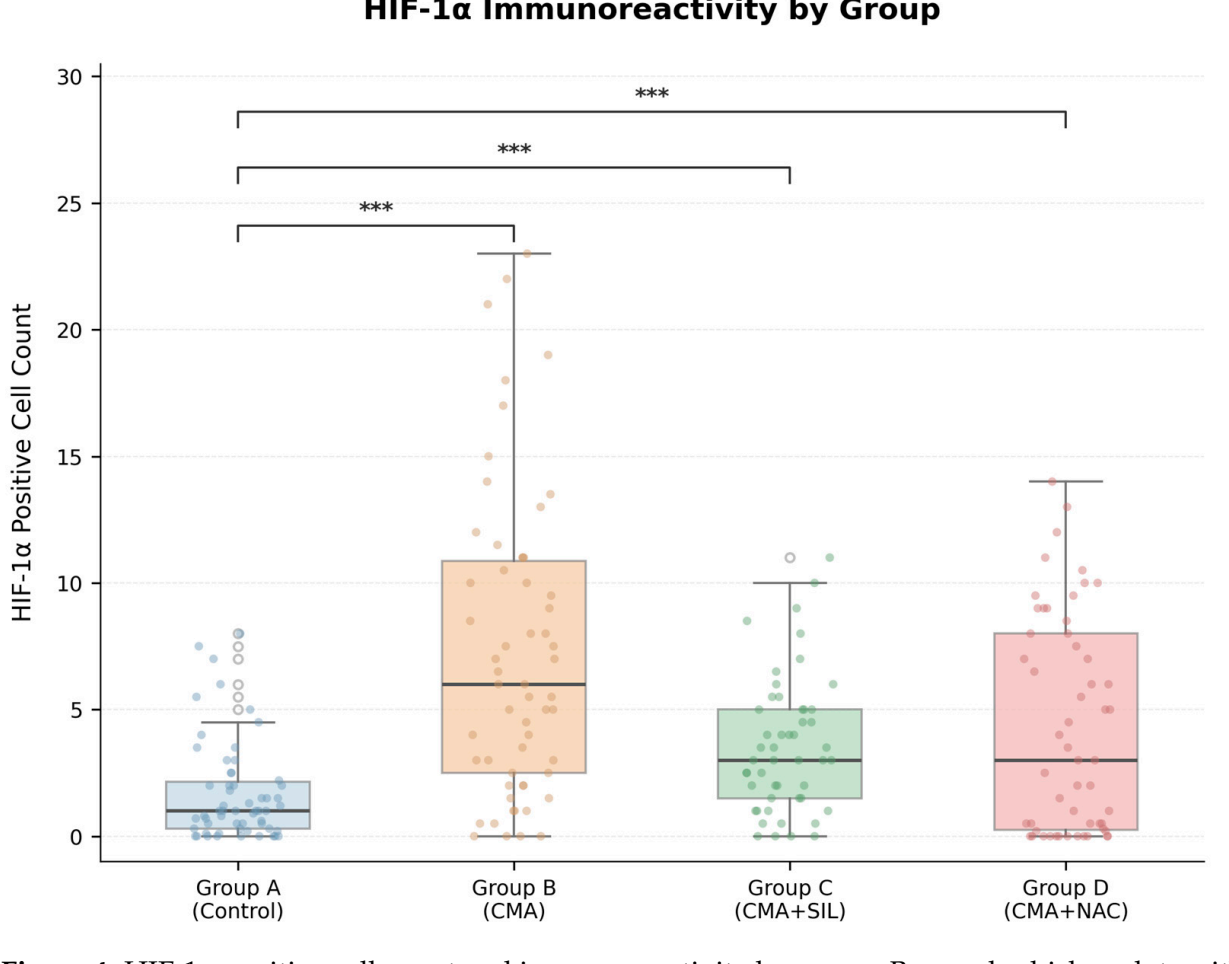
HIF-1α positive cell count and immunoreactivity by group. Box-and-whisker plots with individual data points for HIF-1α IHC-IPC across experimental groups. *n* = 90 fields per group (10 fields × 9 animals). Boxes: IQR; horizontal lines: median; whiskers: 1.5 × IQR. Significance brackets: Dunn’s test with Bonferroni correction (*** *p* < 0.001).

The findings of HIF-1α immunoreactivity are presented in Figure 4. In the control group (Group A), HIF-1α-positive cell count was detected at minimal levels. In the CMA-administered group (Group B), a marked increase in HIF-1α expression was observed, which was statistically significant compared to the control group (*p* < 0.001). In the sildenafil-treated group (Group C, CMA + SIL), HIF-1α positive cell count showed a decrease compared to the CMA group. In the NAC-treated group (Group D, CMA + NAC), HIF-1α expression remained at levels similar to Group B. Multiple comparisons performed using Dunn’s test with Bonferroni correction revealed statistically significant differences between Group A and Group B, Group A and Group D, and Group B and Group C (*p* < 0.001).

## 4. Discussion

### 4.1. Neuroprotective Mechanisms

Both NAC and sildenafil attenuated contrast-induced changes in HIF-1α immunoreactivity, though through potentially different mechanisms. NAC primarily functions as a direct antioxidant and glutathione precursor, mitigating oxidative stress [14,15,16,17]. However, the incomplete protection observed in the CMA + NAC group suggests that antioxidant treatment alone may be insufficient for comprehensive neuroprotection. Sildenafil, a selective PDE5 inhibitor, is known to modulate the NO/cGMP/PKG signaling cascade, with downstream effects on apoptotic pathways, blood–brain barrier integrity, and neuroinflammation as demonstrated in prior preclinical studies [7,8,9,11,12,13]. While these mechanisms provide a plausible pharmacological basis for the trend toward attenuation of HIF-1α immunoreactivity observed in the CMA + SIL group, it must be emphasized that none of these molecular intermediates were directly measured in the present study. The mechanistic interpretation therefore remains speculative and should be tested in future studies incorporating pathway-specific assays.

### 4.2. HIF-1α as a Biomarker

The divergence between ELISA and IHC results warrants careful interpretation. While IHC provides spatial resolution of HIF-1α expression at the cellular level, ELISA captures total protein concentration in tissue homogenate. One hypothesis is that sildenafil and NAC may modify the subcellular distribution or post-translational processing of HIF-1α, though this was not directly assessed in the present study and remains speculative. These observations suggest that HIF-1α immunoreactivity may have value as a biomarker for evaluating neuroprotective interventions, though further validation with quantitative protein assays is needed [16,17].

The divergence between ELISA and IHC findings may be attributed to several factors operating in both directions. ELISA measures total protein in tissue homogenate and is susceptible to HIF-1α degradation during sample processing, potentially underestimating true tissue concentrations. Conversely, IHC-based semi-quantitative scoring is inherently observer-dependent, and the cellular-level resolution that constitutes its strength also introduces vulnerability to field selection bias and inter-rater variability. Formal inter-rater reliability assessment for the IHC scoring was not performed in this study, which is a limitation. Furthermore, the field selection, while described as random, was not conducted using a systematic random sampling protocol. Accordingly, the discrepancy may reflect limitations inherent to either or both assay modalities, and neither result should be interpreted in isolation. Similar discrepancies between ELISA and IHC have been reported in the literature.

### 4.3. Clinical Consequences

The trend toward attenuation of HIF-1α immunoreactivity in the sildenafil-treated group, although not reaching statistical significance after correction for multiple comparisons (*p* = 0.050), raises the possibility that phosphodiesterase-5 inhibitors warrant further investigation as candidate protective agents for contrast-enhanced imaging in high-risk patients (elderly, cerebrovascular disease, renal impairment, hypertension). Sildenafil exhibits a broader scope of action by modulating multiple molecular targets, which may contribute to its greater neuroprotective efficacy compared to single-target agents; for example, it can improve blood flow to the brain, activate pro-survival pathways, alter HIF-1α signaling, and lower neuroinflammation. Subsequent research is required to examine dose–response relationships, optimal timing, synergistic effects with other medicines, functional outcomes, and clinical translation via carefully structured trials [7,8,9,18,19].

Recent clinical reports have further highlighted the clinical significance of contrast-induced encephalopathy across diverse patient populations, including those with chronic kidney disease and following endovascular procedures [2,4]. These reports underscore the urgency of identifying neuroprotective strategies, as the pathophysiology of contrast-induced brain injury remains incompletely understood. Consistent with our findings, recent experimental evidence supports the neuroprotective role of sildenafil through modulation of HIF-1α and related signaling pathways [13,16,17], while the antioxidant properties of NAC continue to be validated in diverse models of oxidative brain injury.

### 4.4. HIF-1α Immunoreactivity

When looking at HIF-1α immunoreactivities, these results show that giving CMA made HIF-1α, an indication of hypoxic stress, more active, whereas giving sildenafil made this increase far less likely. HIF-1α is a key transcription factor that stays stable when there is not enough oxygen and is very crucial for cells to adapt to low oxygen levels [23,24].

Sildenafil, a phosphodiesterase-5 (PDE5) inhibitor, is believed to indirectly influence HIF-1α expression by enhancing tissue oxygenation and diminishing oxidative stress. The substantial decrease in HIF-1α immunoreactivity noted in the sildenafil-treated cohort indicates that sildenafil may mitigate oxidative stress-related cellular damage.

Conversely, NAC therapy did not significantly diminish HIF-1α immunoreactivity relative to the CMA group, despite its recognized antioxidant characteristics. This finding indicates that the control of HIF-1α is not exclusively reliant on oxidative stress [25].

### 4.5. Limitations of the Study

First, the primary outcome measure (tissue HIF-1α by ELISA) did not demonstrate statistically significant group differences (*p* = 0.119), while the secondary semi-quantitative IHC measure did (*p* < 0.001). This discrepancy may reflect limitations of either or both assay modalities, as discussed in Section 4.2.

Second, this study was conducted exclusively in female Wistar rats. Potential sex-based differences in neurotoxic susceptibility, HIF-1α regulation, and neuroprotective responses cannot be excluded, and future studies incorporating both sexes are warranted.

Third, only a single time point (48 h post-contrast) was examined. While consistent with established experimental models, this design does not allow characterization of the temporal dynamics of HIF-1α expression. Future studies incorporating early (24 h) and late (7–14 days) assessments are needed.

Fourth, functional neurological outcomes and behavioral assessments (e.g., Morris Water Maze, rotarod test) were not performed, limiting translational relevance.

Fifth, the mechanistic pathways discussed (NO/cGMP/PKG signaling) were not directly measured. These interpretations are based on published literature and remain speculative.

Sixth, dose–response relationships were not characterized, and the potential confounding effects of using two different anesthetic regimens (pentobarbital for CMA induction; ketamine/xylazine for tissue collection) on HIF-1α expression cannot be excluded.

Seventh, the histopathological findings (H&E) were assessed qualitatively without a standardized semi-quantitative scoring system, limiting the objectivity and reproducibility of these observations.

Finally, inter-rater reliability for IHC scoring was not formally assessed, and field selection was not performed using systematic random sampling protocols, which may introduce observer-dependent bias.

## 5. Conclusions

This study investigated the potential neuroprotective effects of NAC and sildenafil against contrast-induced neurotoxicity in a rat model. The primary outcome measure, tissue HIF-1α concentration by ELISA, did not differ significantly among groups. Secondary semi-quantitative IHC analysis revealed that contrast media administration significantly increased HIF-1α immunoreactivity compared to control, with a large overall effect size. The sildenafil-treated group showed a trend toward lower HIF-1α immunoreactivity compared to the contrast media group, with a medium effect size (Cohen’s d = 0.71), though this difference did not reach statistical significance after correction for multiple comparisons (Dunn’s test, *p* = 0.050). No significant difference was observed between the NAC-treated and untreated contrast media groups.

These findings should be interpreted as preliminary and hypothesis-generating. HIF-1α immunoreactivity warrants further investigation as a potential biomarker for contrast-induced neural injury, and the observed trend toward neuroprotection by sildenafil requires confirmation in larger, adequately powered studies incorporating quantitative protein assays, functional neurological outcomes, and pathway-specific molecular analyses.

## Figures and Tables

**Table 1:** HIF-1α levels and immunohistochemical findings. IHC-IPC: immunohistochemical staining of immunopositive cells. The results are presented as mean ± SD.

Variable	Group A (Control)	Group B (CMA)	Group C (CMA + NAC)	Group D (CMA + SIL)	*p*-Value
Tissue HIF-1α (ng/mL)	0.096 ± 0.056	0.80 ± 0.27	0.174 ± 0.163	0.086 ± 0.042	0.121
IHC-IPC (cells/HPF)	0.72 ± 1.09	7.33 ± 6.16	5.84 ± 4.12	3.91 ± 2.83	<0.001 *

**Note:** The asterisk (*) indicates statistical significance at the *p* < 0.001 level.

## Data Availability

The datasets generated and analyzed during the current study are available from the corresponding author upon reasonable request. Requests for data access should be directed to the corresponding author. The data supporting the findings include raw ELISA measurements, immunohistochemistry scoring data, and statistical analysis outputs.
